# Expert commentary on the challenges and opportunities for surgical site infection prevention through implementation of evidence-based guidelines in the Asia–Pacific Region

**DOI:** 10.1186/s13756-021-00916-9

**Published:** 2021-04-01

**Authors:** K. Morikane, P. L. Russo, K. Y. Lee, M. Chakravarthy, M. L. Ling, E. Saguil, M. Spencer, W. Danker, A. Seno, E. Edmiston Charles

**Affiliations:** 1 Division of Clinical Laboratory and Infection Control, Yamagata University Hospital, Yamagata, Japan; 2 School of Nursing and Midwifery, Monash University, Frankston, VC Australia; 3 Department of Surgery, KyungHee University Medical Center, Seoul, South Korea; 4 Fortis Healthcare Limited, Gurgaon, Haryana India; 5 Infection Prevention and Epidemiology, Singapore General Hospital, Singapore, Singapore; 6 Philippine General Hospital, Manila, Philippines; 7 Infection Prevention Consultant, Boston, MA USA; 8 Ethicon, Johnson and Johnson Medical Device Companies, Somerville, NJ USA; 9 Johnson and Johnson Medical Asia Pacific, Singapore, Singapore; 10 Department of Surgery, Medical College of Wisconsin, Milwaukee, WI USA

**Keywords:** Surgical site infection, Healthcare-associated infection, Guidelines, Implementation, Asia–Pacific

## Abstract

**Introduction:**

Surgical site infections (SSIs) are a significant source of morbidity and mortality in the Asia–Pacific region (APAC), adversely impacting patient quality of life, fiscal productivity and placing a major economic burden on the country’s healthcare system. This commentary reports the findings of a two-day meeting that was held in Singapore on July 30–31, 2019, where a series of consensus recommendations were developed by an expert panel composed of infection control, surgical and quality experts from APAC nations in an effort to develop an evidence-based pathway to improving surgical patient outcomes in APAC.

**Methods:**

The expert panel conducted a literature review targeting four sentinel areas within the APAC region: national and societal guidelines, implementation strategies, postoperative surveillance and clinical outcomes. The panel formulated a series of key questions regarding APAC-specific challenges and opportunities for SSI prevention.

**Results:**

The expert panel identified several challenges for mitigating SSIs in APAC; (a) constraints on human resources, (b) lack of adequate policies and procedures, (c) lack of a strong safety culture, (d) limitation in funding resources, (e) environmental and geographic challenges, (f) cultural diversity, (g) poor patient awareness and (h) limitation in self-responsibility. Corrective strategies for guideline implementation in APAC were proposed that included: (a) institutional ownership of infection prevention strategies, (b) perform baseline assessments, (c) review evidence-based practices within the local context, (d) develop a plan for guideline implementation, (e) assess outcome and stakeholder feedback, and (f) ensure long-term sustainability.

**Conclusions:**

Reducing the risk of SSIs in APAC region will require: (a) ongoing consultation and collaboration among stakeholders with a high level of clinical staff engagement and (b) a strong institutional and national commitment to alleviate the burden of SSIs by embracing a safety culture and accountability.

## The landscape of surgical site infections—a global perspective

Surgical site infections (SSIs) are defined as infections that occur at or near the surgical incision within 30 days following a procedure, or within 90 days if prosthetic materials are implanted at surgery [1]. Reported SSI rates vary according to the type of surgery, surgery length, surgical method (clean vs clean contaminated vs dirty; robotic vs conventional), size of facility (number of beds), and patient cohort age group [2–9]. SSI is one of the most frequently reported types and is the most common postoperative complication [2, 4, 10–12]. SSIs are associated with a poorer post-operative recovery, increased postoperative morbidity and mortality, and SSIs contribute to the spread of antibiotic resistance globally [13]. SSIs remain a significant global health problem that warrants prioritized efforts for *prevention* [14].

The incidence of SSI is difficult to establish because criteria are not always standardized [15]. A substantial proportion of infections is only detected post-discharge and may be treated in the community without notification [13]. According to the World Health Organization (WHO), the cumulative incidence of SSI is 0.9% in the USA (2014) and ranges between 0.75 and 9.5% across different types of surgery in Europe (2010–2011) [2, 16]. However, low-income countries carry a disproportionately greater SSI burden [3, 4, 9, 16–18]. The WHO reports a pooled SSI incidence in low- and middle-income countries (LMICs) of 11.8% [17]. In the APAC region, the reported incidence of SSI varies widely—cumulative incidences of 2.8% in Australia (2002–2013), 2.0–9.7% in the Republic of Korea, 4% in China (2000–2017), and 7.8% in South East Asia and Singapore (pooled; 2000–2012) [3, 17, 19–21].

## The landscape of surgical site infections—an APAC perspective

On July 30–31, 2019 an SSI Prevention Symposium was held in Singapore where 10 stakeholders from across the region and North America representing infection control, surgical sciences, quality services and nursing met to discuss the need for the standardization of evidence-based practices to improve the surgical outcomes of patients in the Asia–Pacific region. The APAC region comprises a diverse range of countries with varying climates and cultural, religious, demographic, and healthcare funding landscapes. The distribution of microbial pathogens responsible for SSIs also varies across the region [16]. As such, APAC presents unique and wide-ranging challenges related to SSI prevention and control within in-patient and post-discharge environments.

While a significant number of recent studies on SSI from the region exists, information on how to utilize the data to improve SSI prevention is scarce. Some countries do not have contextually applicable national guidelines, and many lack standardized protocols and accountabilities for guideline implementation and SSI monitoring. Although some countries have mandatory requirements for SSI reporting that inform reimbursement decisions, several countries within APAC have inadequate infection prevention and control surveillance programs, standardized surveillance systems and accountabilities [3, 16]. SSI rates have been shown to strongly correlate with the degree of concordance to guidelines, and a large systematic review has estimated that half of all SSIs could be prevented through appropriate application of evidence-based strategies [22, 23]. The consensus expert panel that met in Singapore agreed that the burden of SSI in the region could be alleviated through implementation of the standardized SSI prevention guidelines and improved surveillance.

The Singapore symposium served as a platform to: (1) identify persistent gaps and barriers to SSI prevention in APAC, (2) discuss evidence-based SSI prevention strategies and solutions that demonstrated improved outcomes, elevating the standard of patient care, and (3) achieve consensus on the best ways to implement SSI prevention and surveillance guidelines across the APAC region.

## Strategy for achieving consensus

Prior to the Symposium, a targeted literature review was performed to explore the evidence in four key areas—guidelines, implementation, surveillance and outcomes—particularly relevant to APAC, and to identify gaps in these areas. The following keywords were used: “surgical site infection”, “infection control”, “infection prevention”, “guidelines”, “implementation”, “surveillance”, “outcomes”, “economic”, “cost” AND “Asia Pacific”, “Asia”, “China”, “India”, Hong Kong”, “Singapore”, “Japan”, “Korea”, “Taiwan”, “Malaysia”, “Indonesia”, “Vietnam”, “Thailand”, “Philippines”, “Australia”, “New Zealand”. The search was limited to English language publications only, dated 2009–June 2019.

Based on this review, key questions on APAC-specific challenges and opportunities for SSI prevention were formulated and discussed during the Symposium, led by the multidisciplinary experts from Australia, India, Japan, Philippines, Singapore, South Korea and the USA. The delegates asked questions during the Symposium via the Pigeonhole Live® platform. The results presented in this manuscript are a result of both a comprehensive targeted literature review and the inputs/recommendations from the expert panel based on their clinical experience.

## Challenges for SSI guideline implementation in the APAC region

Several countries in APAC have national guidelines, including the Australian Guidelines for the Prevention and Control of Infection in Healthcare, the Chinese guideline for the Prevention of Surgical Site Infection, the Indian Council of Medical Research guideline, the Thailand Surgical Infection Society guidelines, and Ministry of Health guidelines from Indonesia, Singapore, Malaysia and the Philippines [24–31]. Japan has no official government SSI guideline; however, two Japanese professional organizations have relevant publications [32, 34]. The expert panel outlined several key recommendations for selecting, adapting and disseminating guidelines (Table 1). The successful application of these key recommendations was demonstrated through the development of the Australian national guidelines (2019), which was based on local context and published online using an interactive ‘living guideline platform’ [24]. Each recommendation is listed with its strength of recommendation, key evidence regarding benefits/harm, rationale and specific suggestions for decision making and implementation.

**Table 1:** Expert panel key recommendations for guideline selection, adaptation and dissemination

Guideline selection, adaptation and dissemination—expert recommendations
1. Systems and governance for SSI prevention and surveillance within APAC should be consistent with global and/or national guidelines. Guidelines should be implemented at both the national and individual hospital level
2. Professional societies within respective countries may also formulate their own guidelines using frameworks provided by global guidelines and should be consistent with their national guidelines. National guidelines should be situationally applicable and adaptable to an institution’s cultural, socioeconomic, clinical, health economic and political context [40]
3. Where possible, local systems and processes for SSI prevention and surveillance should be developed and standardised based on local evidence to ensure contextual relevance and long-term sustainability
4. Guidelines should be actively disseminated to stakeholders who are in a position to make impactful changes
5. Guidelines should be ‘living documents’, updated on an ongoing basis as new evidence is accumulated

Non-concordance with SSI prevention guidelines was strongly correlated with SSI rate [22]. A key challenge for SSI prevention in the APAC region is achieving widespread and consistent guideline implementation. In many APAC countries, both developed and developing, recent data on guideline adherence and implementation of tools such as SSI bundles are lacking. During the meeting, the expert panel identified several common institutional and individual challenges that could hinder the successful implementation of evidence-based SSI guidelines within the APAC region.

1. *Human resource constraints* Hospitals invariably battle time, workforce and workload constraints, leaving little time or priority for adequate training and protocol modifications. A lack of SSI prevention education among healthcare workers, high nursing staff turnover, limitations in language competency, and even a lack of data interpretation skills can hamper efforts to implement systems that are congruent with the guidelines [16, 34, 35].
2. *Lack of adequate policies and procedures* Absence of, or poor knowledge concerning written policies, checklists or care bundles can negatively affect guideline implementation [36, 37]. Some government health authorities in the APAC region do not have adequate laws or policies to encourage SSI prevention and control.
3. *Lack of strong safety culture* The panel emphasized that cultivation of a strong, sustainable institutional safety culture is imperative when working toward SSI prevention [38]. Obstacles detracting from a safety culture include complacency, a lack of interest or skills/education among staff, self-protective attitudes, or resistance to changing established behaviors [38–41]. Studies have shown that effective communication and teamwork in the operating room are associated with fewer errors [42]. Good administrative support is also essential in establishing a safety culture. The leadership of senior surgeons is critical to the success of SSI prevention planning. Initiatives are more likely to gain traction with active involvement from senior leadership – particularly surgeons – who are prepared to champion the development and implementation of guidelines and influence a culture shift toward process improvement [38].
4. *Limitations in funding and other resources* Reimbursement is an important consideration for medical and surgical practice patterns in the APAC region [16, 43]. A wide spectrum of funding scenarios exists across the region, and inadequate levels of dedicated financial support and infrastructure in some countries is a common barrier to the successful implementation of SSI guidelines. Some low-income countries may also lack the necessary microbiological and diagnostic tools and laboratory resources.
5. *Cultural considerations* Varying cultural norms exist across APAC, some of which may limit the applicability of selective guideline components. For example, in India and Japan, hospital stays can often be long by patient choice. Clipping of hair is culturally less acceptable in some countries. Cultural tendencies toward smoking may also increase SSI risks.
6. *Poor patient awareness and responsibility* Suboptimal patient compliance or thoroughness regarding preoperative self-showering/bathing can interfere with even the best SSI prevention efforts. In addition, in some areas, there may be a lack of awareness among patients and families regarding post-operative care instructions, increasing the risk of SSI following discharge [16].

## Strategies for guideline implementation: an expert panel recommendation

**Fig. 1:**
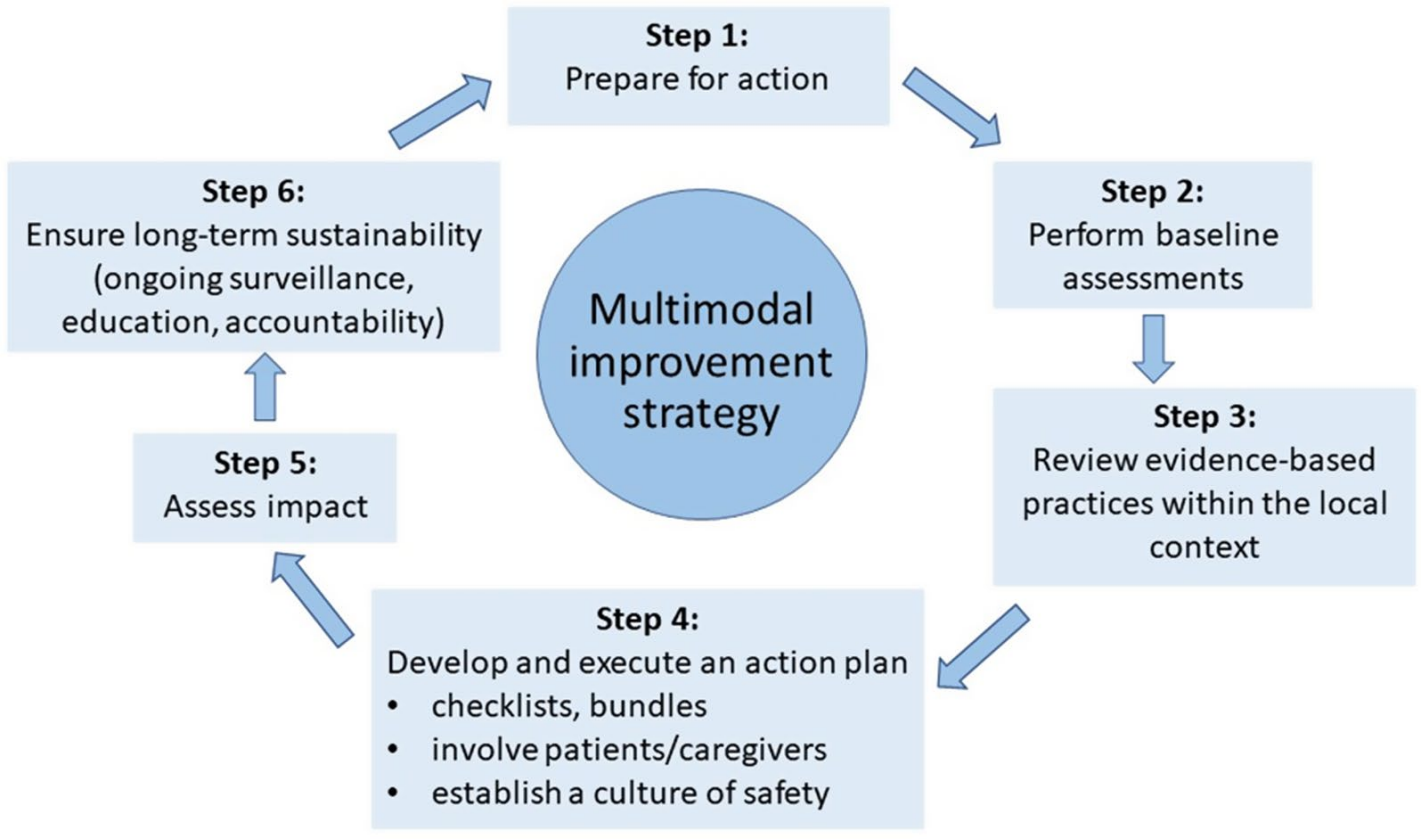
Multimodal SSI prevention and control improvement strategy. Expert panel assessment of multimodal SSI prevention and control improvement strategy. Adapted from: World Health Organization. Implementation manual to support the prevention of surgical site infections at the facility level—turning recommendations into practice (interim version) [13]

The expert panel recommends that every hospital works actively toward improving the implementation of applicable guidelines, through embedding appropriate SSI prevention programs within their work processes. Such changes should be situationally appropriate, measurable and sustainable over the long term. Regardless of the current level of SSI prevention initiatives, we suggest a stepwise approach based on the WHO’s ‘cycle of continuous improvement’ (Fig. 1) [13]. Moving forward, the expert panel has formulated the following evidence-based recommendations.

1. Prepare for action
  Hospitals should systematically identify core principles and relevant key questions that can prompt action and drive infection control as a top priority [24]. Establishing a surgical wound task force can be helpful to gain important insights into local infection cause and prevention, and to facilitate relationships, discussion and understanding. Teams should meet regularly and include all relevant disciplines, including surgeons, surgical nurses, anesthetists, pharmacists, and managers from Infection Prevention and Control (IPC), Healthcare Quality, Facilities, and Environmental Services [44]. It will be crucial to gain buy-in from senior surgeons who are prepared to champion the cause of SSI guideline implementation and disseminate important information; infection prevention and control teams need to proactively build trust with surgeons and place them at the forefront of discussions [45]. Surgeons are most likely to become engaged when presented with robust evidence-based data on SSI rates, compliance with processes and guidelines, antibiotic resistance and appropriate stewardship, and hand hygiene compliance. Surgeons need to be aware of their own SSI data and how these compare with those from other institutions and currently established benchmarks. Some institutions in the region, such as India, encourage surgeons and their teams by recognizing their SSI prevention work with awards or other public recognition, or showcasing their efforts to modify practices and behavior.
2. Perform baseline assessments
  A robust data collection system is crucial to the success of reducing the risk of SSIs. For countries or institutions currently without adequate and proactive SSI prevention systems, the expert panel recommends initially performing a gap analysis or point prevalence survey to assess specific process indicators. For example, current adherence to operating room preparation or postoperative dressing change protocols can be measured and recorded as a baseline against which improvement can be quantified. The key is to start small, evaluate specific indicators of interest, collect small groups of data and tackle a few issues at a time. Systematic tools for the assessment of infection control exist, including a WHO Electronic Assessment Tool, the US Infection Control Assessment Tool (ICAT), and the IPC Assessment Framework (IPCAF) [36, 46–48]. These tools can assist in clearly identifying targets for cost-effective system improvements, including in low-resource facilities.
3. Review evidence-based practices within the local context
  A thorough review of available guidelines will help identify improvements that are most urgent, necessary and cost-effective. For highly cost-sensitive economies and smaller hospitals, improvements must be made within current financial constraints. However, the expert panel notes that it is important to consider real overall costs—e.g., what are the projected overall long-term *savings* from investing in new equipment (e.g. electronic hand hygiene alert systems at or near the hand rub), employing more SSI prevention staff, or using antimicrobial sutures?
4. Develop and execute an action plan
  Efforts should be made to implement guidelines using clearly defined strategies that consider expert consensus, country experience and the specific needs and goals of the facility. To build a system change, it may be useful to initially choose four or five evidence-based practices, standardize interventions into well-defined tasks to facilitate uptake, and establish key performance indicators to monitor improvement.
5. Use of standardized, measurable outcome tools
  Several practical tools to support guideline implementation and best practice are available, including implementation guides, checklists, surgical bundles, risk stratification tools, and standardized protocols. The expert panel recommends careful consideration of how these tools can best inform local practice.
  - Implementation guides
    Along with its global guidelines for SSI prevention, the WHO has published two useful documents to aid in their practical application: an implementation manual and a practical document outlining implementation approaches [13, 49, 50].
  - Checklists
    Use of surgical safety checklists can successfully draw attention to SSI prevention. Patients exposed to a checklist have a lower risk of postoperative infection and death than patients not exposed to a checklist, although this finding could also simply reflect the quality of care in hospitals where checklists are routinely used [51–53]. The global WHO surgical safety checklist is designed to support SSI prevention at the facility level. This checklist comprises 19 items in 3 domains (before anesthesia, before incision, and before the patient leaves the operating room). It has been widely adopted and adapted worldwide and is often considered a surrogate marker for the quality of patient care [53, 54]. Implementation of the WHO checklist has also been evaluated in several APAC countries, including Thailand, India, Cambodia, Pakistan and Indonesia. Variability in awareness and compliance has been highlighted, as well as differences in subjective clinical decision making and cultural norms [51, 55–58].
  - Surgical bundles
    The ‘bundle’ approach has become a commonly accepted and effective method of incorporating best practice measures into routine clinical care [59, 60]. National guidelines from India, Indonesia, Singapore and Australia describe and recommend the use of a bundle approach [24, 26, 28, 29]. No single SSI prevention bundle, however, can be applied to all settings. Bundles are built from usually three to five specific high-quality evidence-based practices (high level of evidence/class 1A; Table 2) [24, 60–62]. Prevention bundles should be constructed based on identified local gaps that are thought to be contributing to higher-than-desirable SSI rates [63]. Consistent use of care bundles has been shown to achieve substantial SSI rate reductions and healthcare cost benefits [64–70]. Use of bundles in APAC countries (e.g. Japan, India, China and Singapore) has been reported to reduce SSI incidence by 22–93% [71–75]. The expert panel strongly recommends that implementation of an evidence-based surgical care bundle be supported by a robust compliance program, since outcome failures following bundle implementation are often associated with poor compliance to individual bundle components [76].
  - Other tools
    The Joint Commission International has designed a ‘toolkit’ document to support the implementation of best practice primarily in the preoperative phase of care, including risk assessment [77]. Other risk stratification tools can also help stratify patients according to SSI risk [78–80].
6. Involvement of patients and caregivers—making all parties part of the solution
  The expert panel recognizes that both the patient and caregiver play a major role in optimizing clinical outcome recommendations. Patients should be fully engaged and empowered with a full understanding of preoperative preparation, as well as infection risk during wound healing [81]. In the Philippine General Hospital, a dedicated pathway (via text messages or email) is established for patients to inform physicians if any problems arise before their designated follow-ups. Patient-reported outcomes (PROs) may be gathered by way of app-based checklists and phone calls. Studies have documented that wound photography can be a useful tool for SSI diagnosis, in addition to chart review and telephone consultation, in improving SSIs diagnostic accuracy and confidence [82–84].
7. Establishing an institutional safety culture
  Implementation of checklists and tools is not likely to impact SSI rates without consistent institution-wide buy-in. A cultural shift toward increased willingness to follow established local protocols drives the effectiveness of checklists and protocols and is strongly associated with improved outcomes [44, 84]. As opposed to enforced mandates which breed only superficial compliance, the preference is to encourage an institutional safety culture wherein staff are impelled, rather than compelled, to be vigilant. Hospitals with a strong organizational safety culture embrace innovation, promote education, collaboration and communication, engage and empower their health professionals, and foster a no-blame, non-punitive climate [85]. Efforts from ‘surgical champions’ to drive the adoption of a safety culture may include modelling safety-focused behavior, making regular safety rounds, initiating daily ‘safety huddles’, direct communication with staff and patients, and participation in quality improvement meetings [38].
8. Assess impact: evaluation and feedback
  The expert panel strongly encourages ongoing evaluation and feedback as crucial aspects of SSI prevention. Feedback of clinical performance data helps create a sense of accountability and motivation [44]. Data-derived surveillance activities are used to identify areas for further practice improvement and research, prioritize action plans, inform policy and practice, and measure the effect of interventions. Regular data feedback to key stakeholders, including frontline providers and hospital leadership, helps support SSI improvement efforts [44]. Demonstrating consistent cost savings—more importantly, overall cost benefits—can help ensure ongoing financial support.
9. Ensure long-term sustainability
  When evidence-based peri- and postoperative practices result in measurable improvement, further steps should be taken to build on this foundation by setting forward new goals. Ongoing education, accountability, and active efforts to engage all stakeholders—particularly where staff turnover is high—can ensure the longevity of SSI prevention programs at a regional, national and APAC-wide level.

**Table 2:** Evidence-based care bundle elements. Adapted from lecture “A Speciality or Global Approach to SSI Prevention: Clean, Clean-Contaminated and Contaminated Procedures” delivered by Dr Charles E Edmiston, Jr., at the APAC Surgical Site Infection Prevention Symposium (Singapore, 31 July 2019)

Bundle elements	Class^a^	Mechanistic benefits
*Evidence-based interventions*		
Normothermia	1A	Less bleeding/preserve immune function in wound bed/enhanced wound healing
Perioperative *weight-based* antimicrobial prophylaxis	1A	Tissue antisepsis/intraoperative conc > MIC [90] wound pathogens
Glycaemic control	1A	Preserve granulocytic immune function/enhance wound healing
Antimicrobial (triclosan) coated sutures (fascia/subcuticular closure)	1A	Mitigate nidus of infection/local tissue antisepsis
Preadmission CHG showering/bathing	1B	Skin antisepsis/reduce skin bioburden
Perioperative skin prep with 2% CHG/70% alcohol	1A	Skin antisepsis/reduce skin bioburden
Separate wound closure tray	II	Mitigate instrument contamination
Glove change prior to fascia/subcuticular closure	II	Disrupt cross-contamination across tissue planes
*Supplemental evidence-based interventions*		
Supplemental oxygen (colorectal surgery)	1A	Enhanced tissue oxygenation and immune function/ metabolic benefits/wound healing
Oral antibiotics/mechanical bowel prep (colorectal surgery)	1A	Reduce bioburden within the bowel lumen and on brush border surfaces
Wound edge protector (colorectal, vascular and OB/GYN surgeries)	1B	Intraoperative wound antisepsis/minimising wound contamination
Staphylococcal decolonization (orthopaedic and CT surgeries)	1A	Mitigate *S. aureus* and MRSA pathogenicity
Smoking cessation (orthopaedic, neurological, CT, and likely all surgeries)	1B	Preserve angiogenesis/reduce risk of dehiscence/enhance wound healing
Intraoperative irrigation of the surgical wound with 0.05% CHG	II	Mitigate wound contamination prior to closure
OR traffic control – minimize door openings	No recommendation/unresolved	Reduce OR air bioburden

CHG, chlorhexidine gluconate; CT, cardiothoracic; MRSA, methicillin-resistant Staphylococcus aureus; OB/GYN, obstetrics/gynaecology; OR, operating room, conc > MIC [90], concentration greater than the minimal inhibitory concentration required to inhibit the growth of 90% of surgical wound pathogens

^a^Column 2: Interventional evidence-based criteria derived from “CDC SSI Guidelines Evidence-Based Criteria documentation and Wisconsin Division of Public Health Service Supplemental Guidance for the Prevention of Surgical Site Infections: An Evidence-based Perspective [60–62]. CDC categories: 1A = strong recommendation supported by high to moderate–quality evidence suggesting net clinical benefits or harms; 1B = strong recommendation supported by low-quality evidence suggesting net clinical benefits or harms or an accepted practice (eg, aseptic technique) supported by low to very low–quality evidence; 1C = A strong recommendation required by state or federal regulation; Category II = weak recommendation supported by any quality evidence suggesting a trade-off between clinical benefits and harms; No recommendation/unresolved issue = An issue for which there is low to very low–quality evidence with uncertain trade-offs between the benefits and harms or no published evidence on outcomes deemed critical to weighing the risks and benefits of a given intervention

## Moving forward—the future is now

The future of SSI prevention globally and in the APAC region lies in ongoing consultation and collaboration between stakeholders, a high level of staff engagement, cultivation of a strong safety culture within institutions and nationally, and forging further research opportunities that can inform ongoing improvements in procedures and systems. The purpose of the Singapore SSI Symposium was to bring together sentinel stakeholders to develop an evidence-based action plan that cuts across the vast geographical, cultural and economic diversity that define the APAC region. Moving forward, the expert panel has identified that further work is needed in the areas of awareness/education, training improvements, audits, simplifying and standardizing practices, and setting achievable standards for accreditation. Ongoing information-sharing regarding effective interventions and differences in epidemiology will be imperative as we work together to reduce SSI risk across the region.

It is also imperative for us to better understand the most effective ways to involve patients and their caregivers in SSI prevention and management. In the future, innovations such as semi-automated or automated surveillance systems, use of vaccines and molecular therapies, implant/suture product developments, and use of dispersion signals and matrix degraders may be employed to reduce SSI risk [86–91]. It is also anticipated that artificial intelligence will be increasingly used to capture surveillance data and will become an integral part of transformative innovation in the future prevention of SSI [92]. Finally, as we have seen during the current pandemic period, telehealth technologies hold a promising future for the implementation and compliance of peri- and postoperative surgical care-processes [93–95].

## Data Availability

No datasets were used in the development of this consensus manuscript.
